# Case of Dislocated Clavicle, Cured in Twenty-Two Days, with Deformity

**Published:** 1838-04-25

**Authors:** 


*Case of Dislocated Clavicle, cured in twenty-two days, with deformity.*

Michael M——, labourer, aged 35, fell, in going down stairs, on the 27th of October, 1837, and struck the extremity of the shoulder. He, however, took little notice of it until the 30th, when, becoming alarmed at the continued stiffness, he consulted a physician; by him it was mistaken for dislocation of the humerus, and sent to the hospital to be reduced. The shoulder was flattened, and apparently hollow near the clavicle, owing to the projection of its scapular extremity; but the rotundity of the shoulder was easily restored by forcing the humerus upwards. He was dressed with the apparatus for fractured clavicle, (mentioned in a previous number,) and placed in bed. As long as he continued perfectly at rest, it remained reduced, but the least motion forced it out again. He was therefore dressed with Desault's bandage, and a large compress over the end of the clavicle; but this did not answer much better; so that, after being confined for some days, all was removed except the sling, the patient being exceedingly restless. In this way, he was allowed to walk, and on the 26th of November was discharged, having the entire use of the arm, although the clavicle still projected a little over the shoulder joint.

